# Inhibition of the ROS-EGFR Pathway Mediates the Protective Action of Nox1/4 Inhibitor GKT137831 against Hypertensive Cardiac Hypertrophy via Suppressing Cardiac Inflammation and Activation of Akt and ERK1/2

**DOI:** 10.1155/2020/1078365

**Published:** 2020-08-04

**Authors:** Si-yu Zeng, Qiu-jiang Yan, Li Yang, Qing-hua Mei, Hui-qin Lu

**Affiliations:** ^1^Department of Pharmacy, Guangdong Second Provincial General Hospital, Guangzhou, Guangdong, China; ^2^Department of Cardiac & Thoracic Surgery, The Third Affiliated Hospital of Guangzhou Medical University, Guangzhou, Guangdong, China; ^3^Laboratory of Vascular Biology, Institute of Pharmacy and Pharmacology, University of South China, Hengyang, Hunan, China; ^4^Institution of Drug Clinical Trial, Guangdong Second Provincial General Hospital, Guangzhou, Guangdong, China

## Abstract

Oxidative stress, inflammation, and hypertension constitute a self-perpetuating vicious circle to exacerbate hypertension and subsequent hypertensive cardiac hypertrophy. NADPH oxidase (Nox) 1/4 inhibitor GKT137831 alleviates hypertensive cardiac hypertrophy in models of secondary hypertension; however, it remains unclear about its effect on hypertensive cardiac hypertrophy in models of essential hypertension. This study is aimed at determining the beneficial role of GKT137831 in hypertensive cardiac hypertrophy in spontaneously hypertensive rats (SHRs) and its mechanisms of action. Treating with GKT137831 prevented cardiac hypertrophy in SHRs. Likewise, decreasing production of reactive oxygen species (ROS) with GKT137831 reduced epidermal growth factor receptor (EGFR) activity in the left ventricle of SHRs. Additionally, EGFR inhibition also reduced ROS production in the left ventricle and blunted hypertensive cardiac hypertrophy in SHRs. Moreover, inhibition of the ROS-EGFR pathway with Nox1/4 inhibitor GKT137831 or selective EGFR inhibitor AG1478 reduced protein and mRNA levels of proinflammatory cytokines tumor necrosis factor *α* (TNF-*α*), interleukin 6 (IL-6), and interleukin 1*β* (IL-1*β*), as well as the activities of Akt and extracellular signal-regulated kinase (ERK) 1/2 in the left ventricle of SHRs. In summary, GKT137831 prevents hypertensive cardiac hypertrophy in SHRs, Nox-deprived ROS regulated EGFR activation through positive feedback in the hypertrophic myocardium, and inhibition of the ROS-EGFR pathway mediates the protective role of GKT137831 in hypertensive cardiac hypertrophy via repressing cardiac inflammation and activation of Akt and ERK1/2. This research will provide additional details for GKT137831 to prevent hypertensive cardiac hypertrophy.

## 1. Introduction

Hypertension is one of the most common cardiovascular diseases and results in heavy burdens worldwide. Chronic pressure overload causes hypertensive cardiac hypertrophy that protects the heart in the early phase; nevertheless, prolonged cardiac hypertrophy leads to cardiac dysfunction, eventually promoting to the origin and development of heart failure. Thus, inhibiting or reversing hypertensive cardiac hypertrophy will contribute to slow down or prevent the progression from hypertension to heart failure.

Chronic pressure overload induces oxidative stress by producing excessive reactive oxygen species (ROS). And sustained oxidative stress has been considered a critical response to sustained high blood pressure and plays an important role in hypertensive cardiac hypertrophy [1]. However, a meta-analysis of seven randomized vitamin E trials does not support the beneficial role of vitamin E therapy in the progression of cardiovascular disease or on clinical events in patients at high risk or with established disease [2]. The failure of classic antioxidants has resulted in a search for new, more effective compounds.

NADPH oxidase (Nox) 2 and Nox4 are major resource for ROS in cardiac cells. Several studies have demonstrated the role of Nox4 in hypertensive cardiac hypertrophy although two contradictory viewpoints emerge from different studies [3–6]. The disparity may be attributed to methodological differences among previous studies, including methodologies for Nox4 overexpression and gene deletion, as well as different kinds and severity of hypertensive models. Nox1 is mainly distributed within the vascular wall and positively mediates doxorubicin-induced cardiac fibrosis [7]. Therefore, inhibiting ROS production by targeting Nox4 and Nox1 might provide better antioxidant therapies to prevent the transition from hypertension to chronic heart failure.

GKT137831 is a small molecule inhibitor of Nox4 and Nox1 with good oral bioavailability and has been shown to prevent hypertensive cardiac hypertrophy in angiotensin II-infused mice with cardiac-specific human Nox4 transgenic mice [5, 8, 9]. Moreover, our previous research also showed that GKT137831 attenuates hypertensive cardiac hypertrophy in rats subjected to abdominal artery constriction (AAC) [10]. These observations indicate that Nox1/4 inhibitor GKT137831 can prevent hypertensive cardiac hypertrophy in models of secondary hypertension. However, it is necessary to elucidate the effect of GKT137831 on cardiac hypertrophy in essentially hypertensive models, such as spontaneously hypertensive rats (SHRs), considering that essential hypertension accounts for at least 90% of all hypertension.

Oxidative stress, inflammation, and hypertension constitute a self-perpetuating vicious circle to exacerbate hypertension and subsequent hypertensive cardiac hypertrophy, thereby accelerating the transition from hypertension to heart failure [11, 12]. Nevertheless, it remains unclear how oxidative stress interacts with inflammation in the hypertrophic myocardium. Thus, the aims of this paper are to further elucidate the effect of Nox1/4 inhibitor GKT137831 on hypertensive cardiac hypertrophy in SHRs and how cardiac inflammation mediates the beneficial role of GKT137831 in hypertensive cardiac hypertrophy.

## 2. Materials and Methods

### 2.1. Ethical Approval

All animal protocols were performed according to the guidelines and principles for the Care and Use of Laboratory Animals issued by the United States National Institutes of Health. Concurrently, these animal protocols were approved by the Medical Ethics Committee of the Guangdong Second Provincial General Hospital.

### 2.2. Materials

Both Nox1/4 inhibitor GKT137831 (DC8118) and selective EGFR inhibitor AG1478 (DC1078) were purchased from D&C Chemicals (Shanghai, China). All antibodies used in the present study were described as follows: p-EGFR antibody (ab5644, Abcam, USA), EGFR antibody (ab52894, Abcam, USA), p-Akt antibody (4060s, Cell Signaling Technology, USA), Akt antibody (2920s, Cell Signaling Technology, USA), p-ERK1/2 antibody (#4695, Cell Signaling Technology, USA), and ERK1/2 antibody (#4370, Cell Signaling Technology, USA).

### 2.3. Animals

Male SHRs (weight 220-250 g) and weight- and sex-matched Wistar Kyoto (WKY) rats were acquired at an age of 12 weeks from Charles River (Beijing, China). After being acclimated for 1 week, SHRs and WKY rats were randomly divided into four groups and two control groups, respectively. All groups in animal experiments with Nox1/4 inhibitor GKT137831 (30 mg/kg day) or selective EGFR inhibitor AG1478 (20 mg/kg day) were the following: control, SHR, and SHR+treatment, each group had 10 rats. GKT137831 was dissolved in vehicle (1.2w% methylcellulose and 0.1w% polysorbate 80 in water) and AG1478 in vehicle (dimethyl sulfoxide). SHRs in the SHR+treatment group were treated with AG1478 via intraperitoneal injection or with GKT137831 through gastric gavage for 4 weeks, while rats in the control group and SHR group were administered the corresponding vehicle.

### 2.4. Measurement of Blood Pressure

Systolic blood pressure (SBP) was measured before the treatment and at the fourth week post the treatment using the tail-cuff method.

### 2.5. Echocardiographic and Hemodynamic Measurements

At the end of the fourth week post the treatment, rats were anaesthetized in ultrasonic atomization with 2% isoflurane. Next, a Vevo 2100 high-resolution in vivo microimaging system (VisualSonics, Canada) was employed to obtain high-quality images used for measuring left ventricular internal diameter during diastole (LVIDd), left ventricular internal diameter during systole (LVIDs), left ventricular anterior wall thickness during diastole (LVAWd), left ventricular anterior wall thickness during systole (LVAWs), left ventricular posterior wall thickness during diastole (LVPWd), left ventricular posterior wall thickness during systole (LVPWs), fractional shortening, and ejection fraction as described previously [10].

After rats were anaesthetized with sodium pentobarbital (i.p., 45 mg/kg), a 24-gauge polyethylene catheter filled with heparin was introduced into the right carotid artery of rats, where aortic systolic pressure (AoSP) and aortic diastolic pressure (AoDP) were measured using a BL-420S system (Chengdu Tai-meng Technology Co., Ltd., Sichuan, China). Subsequently, the maximal rate of the left ventricular pressure increase (*dp*/*dt* max) and decrease (*dp*/*dt* min) and heart rate were determined after the catheter was further introduced into the left ventricle.

### 2.6. Histological Analysis

Arrested in diastole using potassium chloride (30 mmol/L), the heart was fixed with 10% formalin overnight and embedded in paraffin. Subsequently, transverse and transmural slices of the left ventricle (about 5 *μ*m thickness) were prepared and stained with hematoxylin and eosin. Finally, myocyte cross-sectional area was measured in 10 randomly chosen nonrepeating fields in cross-sections stained with hematoxylin and eosin using ImagePro Plus 6.0 according to the method previously described [10, 13]. Furthermore, interstitial fibrosis was quantified as the percentage of fibrotic area over the total myocardial area in 5 randomly chosen nonrepeating visual fields of sections stained with Masson's trichrome reagent using ImagePro Plus 6.0.

### 2.7. Immunohistochemistry

The protein levels of collagen I (Col I) and collagen III (Col III) in the left ventricle were detected using immunohistochemistry as described previously [10].

### 2.8. Real-Time Quantitative PCR

RNA extraction and real-time quantitative PCR were performed as described previously [14]. Real-time PCR used primers for atrial natriuretic peptide (ANP), brain natriuretic peptide (BNP), tumor necrosis factor *α* (TNF-*α*), interleukin 1*β* (IL-1*β*), interleukin 6 (IL-6), collagen I (Col I), and collagen III (Col III), and the gene-specific GADPH was employed as an inner control. The primers were described as follows: ANP: 5′-GGAAGTCAACCCGTCTCA-3′ (forward primer) and 5′-AGCCCTCAG TTTGCTTTT-3′ (reverse primer); BNP: 5′-ATGCAGAAGCTGCTGGAGC TGATA-3′ (forward primer) and 5′-TTGTAGGGCCTTGGTCCTTTGAGA-3′ (reverse primer); TNF-*α*: 5′-TGGCGTGTTCATCCGT TCTC-3′ (forward primer) and 5′-CCCAGAGCCACA ATTCCCTT-3′ (reverse primer); IL-1*β*: 5′-TCCTCTGTGACTCGTGGGAT-3′ (forward primer) and 5′-TCAGACAGCACGAGGCATTT-3′ (reverse primer); IL-6: 5′-TCCTACCCCAACTTCC AATGCTC-3′ (forward primer) and 5′-TTG GATGGTCTTGGTCCTTAGCC-3′ (reverse primer); Col I: 5′-GCCTCAAGGTATTGCTGGAC-3′ (forward primer) and 5′-ACCTTGTTTGCCAGGT TCAC-3′ (reverse primer); Col III: 5′-CTGGACCCCAGGGTCTTC-3′ (forward primer) and 5′-CATCTGATCCAGGGTTTCCA-3′ (reverse primer); GADPH: 5′-ATCAAGAAGGTGGTGAAG CA-3′ (forward primer) and 5′-AAGGTGGAAGAATGGGAGTTG-3′ (reverse primer).

### 2.9. Enzyme-Linked Immunosorbent Assay (ELISA)

The protein levels of proinflammatory factors (such as TNF-*α*, IL-1*β*, and IL-6) in the left ventricle were measured through ELISA as described previously [14].

### 2.10. Detection of Hydrogen Peroxide (H_2_O_2_) and Malondialdehyde (MDA)

H_2_O_2_ and MDA levels were separately detected using the hydrogen peroxide assay kit (S0038) and lipid peroxidation MDA assay kit (S0131) that were purchased from Beyotime Biotechnology (Shanghai, China). Procedures were the following: firstly, a standard curve was constructed using different H_2_O_2_ or MDA solutions and corresponding values of optical density; secondly, samples obtained from the left ventricle were prepared using cell lysis buffer and then used to detect corresponding values of optical density under 520 nm; and thirdly, H_2_O_2_ and MDA levels were calculated using the standard curve and values of optical density.

### 2.11. Western Blotting

Western blotting was performed according to standard procedures previously described [15].

### 2.12. Statistical Analysis

Data are expressed as mean ± standard deviation. Statistical analyses were carried out using the unpaired *t*-test between two groups and one- or two-way ANOVA followed by Bonferroni's post hoc test among at least three groups, and *P* < 0.05 was considered to have statistical significance.

## 3. Results

### 3.1. Nox1/4 Inhibitor GKT137831 Inhibited Hypertensive Cardiac Hypertrophy in SHRs

**Figure 1:**
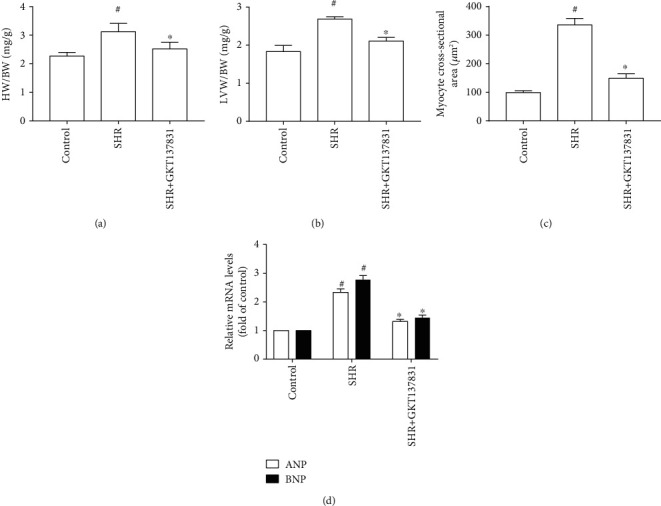
Nox1/4 inhibitor GKT137831 alleviated hypertensive cardiac hypertrophy in spontaneously hypertensive rats. (a) HW/BW (*n* = 6 per group). (b) LVW/BW (*n* = 6 per group). (c) Myocyte cross-sectional area (*n* = 4 per group). (d) mRNA levels of ANP and BNP in the left ventricle (*n* = 4 per group). SHR represents spontaneously hypertensive rats, HW/BW represents the ratio between heart weight and body weight, and LVW/BW represents the ratio between left ventricular weight and body weight. ^#^*P* < 0.05 vs. the control group; ^∗^*P* < 0.05 vs. the SHR group.

**Figure 2:**
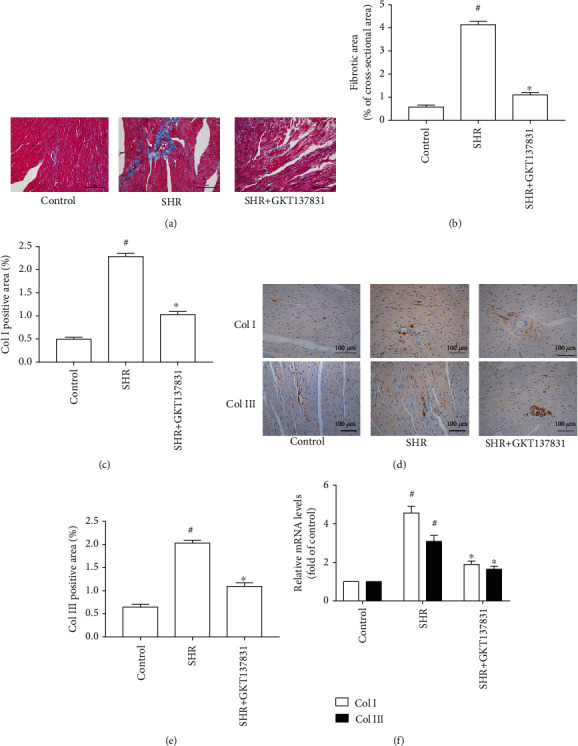
Nox1/4 inhibitor GKT137831 inhibited cardiac fibrosis in spontaneously hypertensive rats. (a) Representative microphotographs of Masson staining of the heart. (b) Fibrosis area (*n* = 4 per group). (c) Col I-positive area (*n* = 4 per group). (d) Representative microphotographs of immunochemistry for Col I and Col III. (e) Col III-positive area (*n* = 4 per group). (f) mRNA levels of Col I and Col III in the left ventricle (*n* = 4 per group). SHR represents spontaneously hypertensive rats, Col I represents collagen I, and Col III represents collagen III. ^#^*P* < 0.05 vs. the control group; ^∗^*P* < 0.05 vs. the SHR group.

Compared with the control group, SBP, AoSP, and AoDP were significantly increased in the SHR group; nevertheless, treatment with GKT137831 failed to reduce SBP, AoSP, and AoDP in SHRs (Table S1 and Table 1). The heart in SHRs exhibited marked hypertensive cardiac hypertrophy, indicated by increases in LVAWs, LVAWd, LVPWs, LVPWd, ratio between heart weight and body weight (HW/BW), ratio between left ventricular weight and body weight (LVW/BW), myocyte cross-sectional area, and mRNA levels of hypertrophic genes (ANP and BNP). By contrast, treating with GKT137831 prevented elevations of LVAWs, LVAWd, LVPWs, LVPWd, HW/BW, LVW/BW, myocyte cross-sectional area, and mRNA levels of hypertrophic genes (ANP and BNP) in SHRs (Figure 1 and Table 1). Moreover, GKT137831 significantly attenuated cardiac fibrosis indicated by the reduction in fibrotic area and the protein and mRNA levels of Col I and Col III (Figure 2). These findings indicated that GKT137831 attenuated hypertensive cardiac hypertrophy independent of blood pressure.

### 3.2. Reducing ROS Production with GKT137831 Inhibited EGFR Activation through Positive Feedback in the Left Ventricle of SHRs

**Figure 3:**
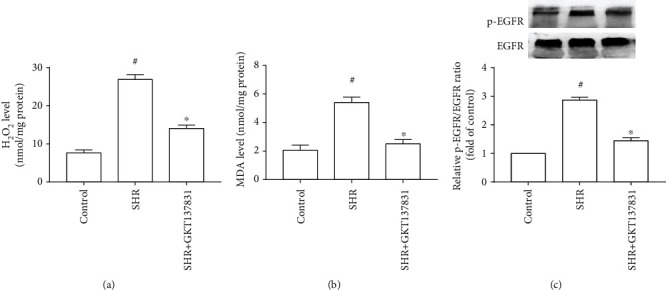
Nox1/4 inhibitor GKT137831 reduced reactive oxygen species (ROS) production and epidermal growth factor receptor (EGFR) activation in the left ventricle of spontaneously hypertensive rats. (a) H_2_O_2_ level (*n* = 4 per group). (b) MDA level (*n* = 4 per group). (c) EGFR activity (*n* = 3 per group). SHR represents spontaneously hypertensive rats; H_2_O_2_ represents hydrogen peroxide; MDA represents malondialdehyde. ^#^*P* < 0.05 vs. the control group; ^∗^*P* < 0.05 vs. the SHR group.

**Figure 4:**
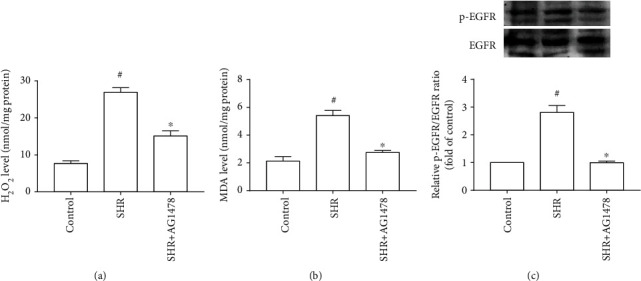
Selective epidermal growth factor receptor (EGFR) inhibitor AG1478 decreased reactive oxygen species (ROS) production in the left ventricle of spontaneously hypertensive rats. (a) H_2_O_2_ level (*n* = 4 per group). (b) MDA level (*n* = 4 per group). (c) EGFR activity (*n* = 3 per group). SHR represents spontaneously hypertensive rats; H_2_O_2_ represents hydrogen peroxide; MDA represents malondialdehyde. ^#^*P* < 0.05 vs. the control group; ^∗^*P* < 0.05 vs. the SHR group.

Among ROS generated in cells within the cardiovascular system, O^2−^ and H_2_O_2_ appear to be very important. Considering that O^2−^ is short-lived because of its rapid transformation to H_2_O_2_ by superoxide dismutase in biological systems [16], H_2_O_2_ content is used to assess ROS production. Moreover, MDA is usually used for evaluating oxidative stress because it is a natural product of lipid peroxidation that occurs when cells are subjected to oxidative stress. Figures 3(a) and 3(b) present the inhibitory effect of Nox1/4 inhibitor GKT137831 on ROS production and MDA level in the left ventricle of SHRs. Subsequently, we observed the effect of GKT137831 on EGFR activation.  Figure 3(c) indicates that GKT137831 diminished EGFR activity in the left ventricle of SHRs. Furthermore, selective EGFR inhibitor AG1478 remarkably decreased EGFR activity, as well as the contents of H_2_O_2_ and MDA in the left ventricle of SHRs (Figure 4). Overall, blocking ROS production with GKT137831 restrained pressure overload-induced EGFR activation via positive feedback in the left ventricle.

### 3.3. EGFR Inhibition Prevented Hypertensive Cardiac Hypertrophy in SHRs

**Figure 5:**
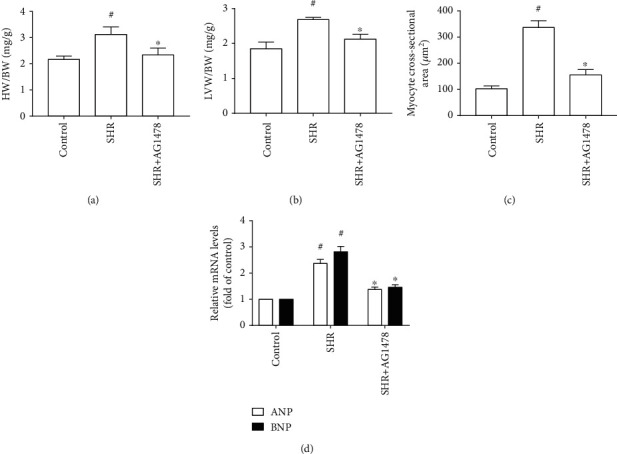
Selective epidermal growth factor receptor (EGFR) inhibitor AG1478 inhibited hypertensive cardiac hypertrophy in spontaneously hypertensive rats. (a) HW/BW (*n* = 6 per group). (b) LVW/BW (*n* = 6 per group). (c) Myocyte cross-sectional area (*n* = 4 per group). (d) mRNA levels of ANP and BNP in the left ventricle (*n* = 4 per group). SHR represents spontaneously hypertensive rats; HW/BW represents the ratio between heart weight and body weight; LVW/BW represents the ratio between left ventricular weight and body weight. ^#^*P* < 0.05 vs. the control group; ^∗^*P* < 0.05 vs. the SHR group.

**Figure 6:**
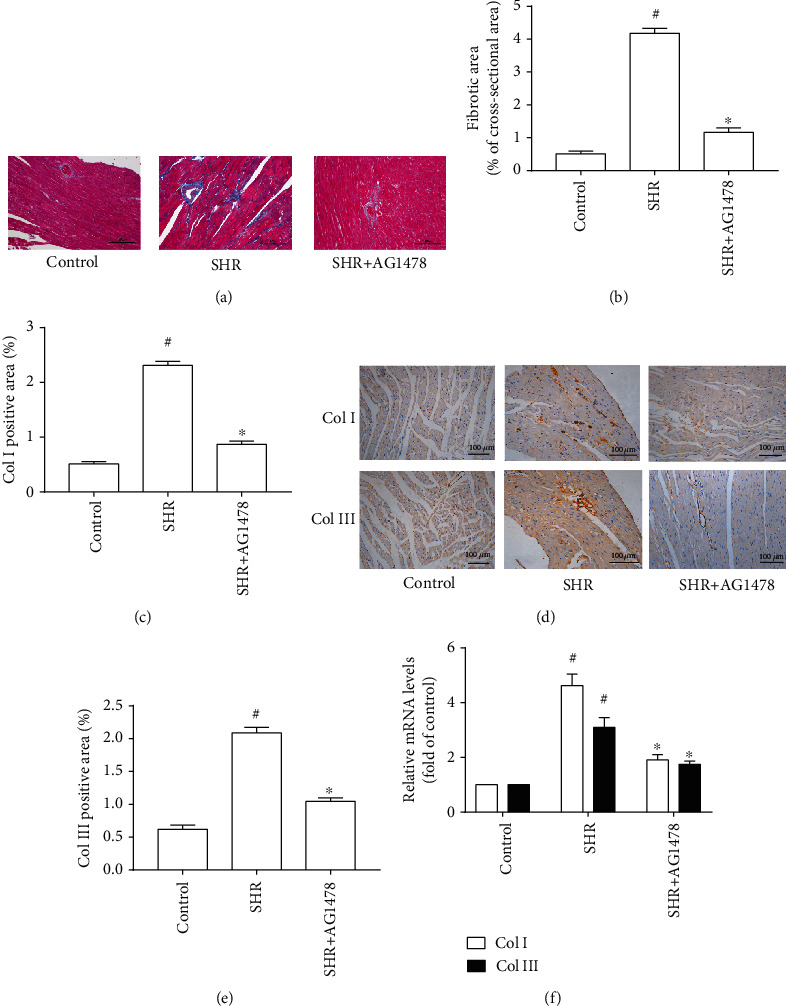
Selective epidermal growth factor receptor (EGFR) inhibitor AG1478 attenuated cardiac fibrosis in spontaneously hypertensive rats. (a) Representative microphotographs of Masson staining of the heart. (b) Fibrosis area (*n* = 4 per group). (c) Col I-positive area (*n* = 4 per group). (d) Representative microphotographs of immunochemistry for Col I and Col III. (e) Col III-positive area (*n* = 4 per group). (f) mRNA levels of Col I and Col III in the left ventricle (*n* = 4 per group). SHR represents spontaneously hypertensive rats, Col I represents collagen I, and Col III represents collagen III. ^#^*P* < 0.05 vs. the control group; ^∗^*P* < 0.05 vs. the SHR group.

Even though treatment with selective EGFR inhibitor AG1478 caused no reduction in SBP, AoSP, and AoDP in SHRs (Table S2), it alleviated hypertensive cardiac hypertrophy, indicated by notable decreases in LVAWs, LVAWd, LVPWs, LVPWd, HW/BW, LVW/BW, myocyte cross-sectional area, and mRNA levels of hypertrophic genes (ANP and BNP) in SHRs (Figure 5 and Table 2). These findings agree with our previous results that AG1478 reduced myocyte cellular area and mRNA levels of hypertrophic genes (ANP and BNP) in primary cardiomyocytes treated with 100 nmol/L angiotensin II for 24 hours [13]. Furthermore, treating with AG1478 caused marked reduction in fibrotic area and the protein and mRNA levels of Col I and Col III (Figure 6). Thus, EGFR inhibition prevented hypertensive cardiac hypertrophy in SHRs independent of blood pressure.

### 3.4. Inhibition of the ROS-EGFR Pathway Reduced Expression of Proinflammatory Cytokines in the Left Ventricle of SHRs

**Figure 7:**
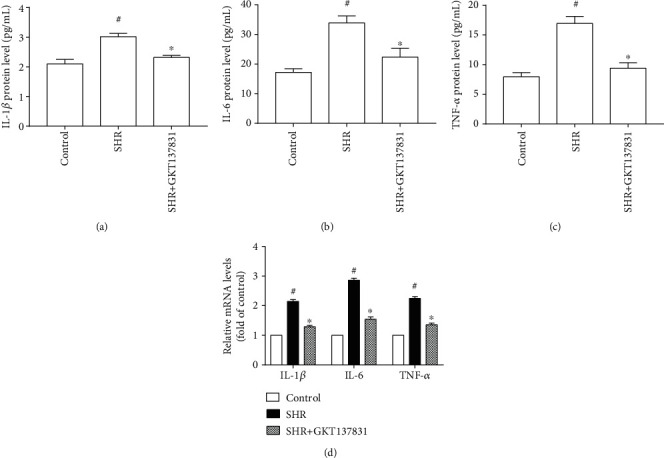
Nox1/4 inhibitor GKT137831 reduced the expression of proinflammatory cytokines in the left ventricle of spontaneously hypertensive rats. (a) IL-1*β* protein level (*n* = 5 per group). (b) IL-6 protein level (*n* = 5 per group). (c) TNF-*α* protein level (*n* = 5 per group). (d) mRNA levels of IL-1*β*, IL-6, and TNF-*α* (*n* = 4 per group). SHR represents spontaneously hypertensive rats, IL-1*β* represents interleukin 1*β*, IL-6 represents interleukin 6, and TNF-*α* represents tumor necrosis factor *α*. ^#^*P* < 0.05 vs. the control group; ^∗^*P* < 0.05 vs. the SHR group.

**Figure 8:**
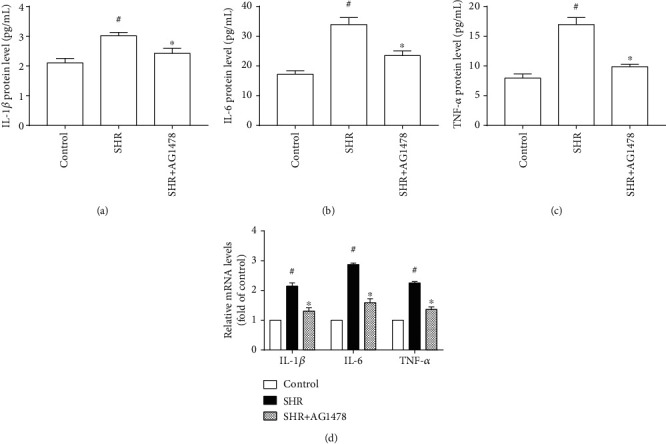
Selective epidermal growth factor receptor (EGFR) inhibitor AG1478 decreased the expression of proinflammatory cytokines in the left ventricle of spontaneously hypertensive rats. (a) IL-1*β* protein level (*n* = 5 per group). (b) IL-6 protein level (*n* = 5 per group). (c) TNF-*α* protein level (*n* = 5 per group). (d) mRNA levels of IL-1*β*, IL-6, and TNF-*α* (*n* = 4 per group). SHR represents spontaneously hypertensive rats, IL-1*β* represents interleukin 1*β*, IL-6 represents interleukin 6, and TNF-*α* represents tumor necrosis factor *α*. ^#^*P* < 0.05 vs. the control group; ^∗^*P* < 0.05 vs. the SHR group.

The expression of proinflammatory cytokines IL-1*β*, IL-6, and TNF-*α* is usually adopted to evaluate inflammation. As shown in Figures 5 and 6, protein and mRNA levels of TNF-*α*, IL-6, and IL-1*β* were upregulated in the left ventricle of SHRs compared with the control group, whereas treating either with Nox1/4 inhibitor GKT137831or with selective EGFR inhibitor AG1478 resulted in significant reductions in the protein and mRNA levels of TNF-*α*, IL-6, and IL-1*β* in the left ventricle of SHRs (Figures 7 and 8). These findings indicate that inhibition of the ROS-EGFR pathway lessened pressure overload-induced cardiac inflammation in SHRs.

### 3.5. The ROS-EGFR Pathway Promoted Akt and ERK1/2 Activation in the Left Ventricle of SHRs

**Figure 9:**
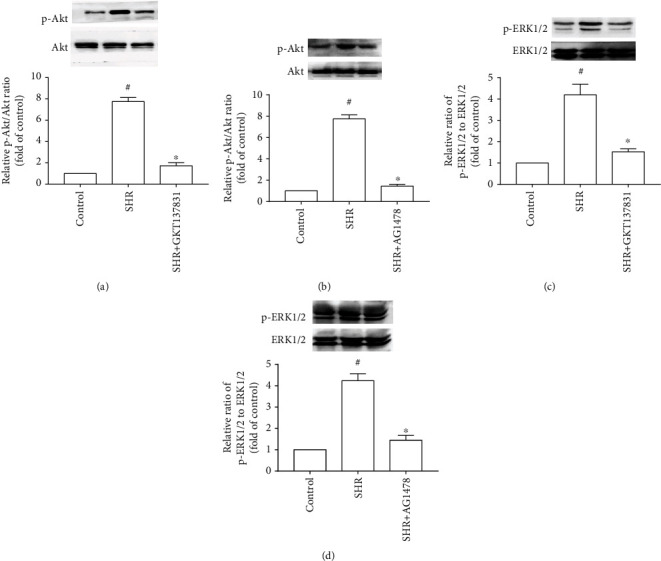
Reactive oxygen species- (ROS-) epidermal growth factor receptor (EGFR) pathway promoted Akt activation in the left ventricle of spontaneously hypertensive rats. (a, b) Effect of Nox1/4 inhibitor GKT137831 (a) or selective EGFR inhibitor AG1478 (b) on Akt activity. (c, d) Effect of GKT137831 (c) or AG1478 (d) on ERK1/2 activity. SHR represents spontaneously hypertensive rats, *n* = 3 per group. ^#^*P* < 0.05 vs. the control group; ^∗^*P* < 0.05 vs. the SHR group.

Figure 7 represents that the activities of Akt and ERK1/2 were remarkably increased in the left ventricle of SHRs compared with the control group, whereas both diminishing ROS production with GKT137831 and inhibiting EGFR with AG1478 caused a significant reduction in the activities of Akt and ERK1/2 in the left ventricle of SHRs (Figure 9). Therefore, the ROS-EGFR pathway promoted pressure overload-induced Akt and ERK1/2 activation in the left ventricle.

## 4. Discussion

In this research, there were three interrelated discoveries: (1) Nox1/4 inhibitor GKT137831 attenuated hypertensive cardiac hypertrophy in SHRs, (2) reducing ROS production with Nox1/4 inhibitor GKT137831 suppressed pressure overload-induced EGFR activation in the left ventricle via positive feedback, and (3) inhibition of the ROS-EGFR pathway mediated pressure overload-induced cardiac inflammation and activation of Akt and ERK1/2 in the left ventricle of SHRs.

A considerable amount of studies in secondary models of hypertension indicate that reducing ROS production with Nox1/4 inhibitor GKT137831 attenuates hypertensive cardiac hypertrophy in rats subjected to abdominal artery constriction and angiotensin II-infused mice with cardiac-specific human Nox4 transgenic mice [5, 10]. In the current study, we further demonstrated the beneficial role of Nox1/4 inhibitor GKT137831 in hypertensive cardiac hypertrophy in a classical model of essential hypertension. These findings suggest that Nox1/4 inhibitor GKT137831 protects against hypertensive cardiac hypertrophy.

EGFR activation is considered to act as a central transducer of heterologous signaling systems, such as those activated by angiotensin II, endothelin, and oxidative stress, all of which can lead to hypertensive cardiac hypertrophy. In vitro experiments indicate that EGFR activation promotes *α*1-adrenergic receptor- and angiotensin II-induced cardiac hypertrophy [13, 17]. Results of in vivo experiments also indicated that EGFR activation advances hypertensive cardiac hypertrophy in angiotensin II-induced hypertensive rats and SHRs [18, 19], in agreement with our present findings that EGFR inhibition inhibited hypertensive cardiac hypertrophy in SHRs. Accordingly, EGFR activation is required in hypertensive cardiac hypertrophy.

Our previous findings showed that Nox4-deprived ROS induces EGFR activation in primary cardiomyocytes [13]. In the present research, decreasing ROS production with Nox1/4 inhibitor GKT137831 caused a significant reduction in EGFR activity in the left ventricle of SHRs. Moreover, EGFR activation also promotes ROS production in the left ventricle of SHRs, in agreement with the results reported by Liang et al. [20] that EGFR inhibition diminishes ROS production in hypertrophic cardiomyocytes of streptozotocin-induced diabetic mice. Collectively, Nox-deprived ROS promotes pressure overload-induced EGFR activation through positive feedback in the hypertrophic myocardium and inhibition of the ROS-EGFR pathway mediates the protective effect of Nox1/4 inhibitor GKT137831 on hypertensive cardiac hypertrophy.

Cardiac inflammation plays an important role in hypertensive cardiac hypertrophy. IL-6 gene deletion inhibits angiotensin II- or transverse aortic constriction- (TAC-) induced hypertensive cardiac hypertrophy, while IL-6 infusion causes left ventricular hypertrophy independent of blood pressure [21–23]. However, Lai et al. [24] reported contradictory findings: IL-6 deletion fails to influence TAC-induced hypertensive cardiac hypertrophy. This discrepancy may be attributed to the methods of disrupting IL-6 gene. TNF-*α* promotes aortic constriction or angiotensin II-induced hypertensive cardiac hypertrophy [25, 26]. Cardiac-specific overexpression of human interleukin 1*α* (IL-1*α*) results in cardiac hypertrophy, and systemic administration of IL-1*β* antibodies or IL-1*β* deletion prevents aortic banding-induced hypertensive cardiac hypertrophy [27–29]. Our present findings indicated that inhibiting the ROS-EGFR pathway with Nox1/4 inhibitor GKT137831 or selective EGFR inhibitor AG1478 markedly reduced the expression of IL-1*β*, IL-6, and TNF-*α* in the left ventricle of spontaneously hypertensive rats. Thus, the ROS-EGFR pathway mediates the effect of Nox1/4 inhibitor GKT137831 on hypertensive cardiac hypertrophy via cardiac inflammation.

Akt activation promotes hypertensive cardiac hypertrophy. Short-term Akt activation improves contractile function in the failing hearts independent of hypertrophy; however, chronic Akt activation induces cardiac hypertrophy by activating mammalian target of rapamycin (mTOR) in transgenic mice with constitutively active Akt [30–32]. Moreover, inhibiting Akt-mTOR signaling with rapamycin ameliorates hypertensive cardiac hypertrophy in spontaneously hypertensive rats and mice with ascending aortic constriction [33, 34]. In the present research, blocking the ROS-EGFR pathway with Nox1/4 inhibitor GKT137831 or selective EGFR inhibitor AG1478 decreased Akt activity in the hypertrophic myocardium of SHRs. Accordingly, inhibition of the ROS-EGFR pathway might mediate the protective action of Nox1/4 inhibitor GKT137831 against hypertensive cardiac hypertrophy by decreasing Akt activity.

ERK1/2 plays a key role in hypertensive cardiac hypertrophy. ERK1/2 inhibition prevented endothelin-1-induced cardiac hypertrophy in cardiomyocytes from spontaneously hypertensive rats and WKY rats [35]. Experiments using transgenic mutation mice at the Thr188 phosphorylation site of ERK2 indicate that ERK1/2 has a causal relationship with cardiac hypertrophy [36]. ERK1/2 deletion in cardiomyocytes blunts hypertensive cardiac hypertrophy in mice with transverse aortic constriction (TAC) [37]. Additionally, MEK1-ERK1/2 promotes cardiac hypertrophy without signs of cardiomyopathy or lethality up to 12 months of age in MEK1 transgenic mice [38]. However, it has been reported that deleting ERK1/2 in the heart did not significantly attenuate angiotensin II-induced hypertensive cardiac hypertrophy [39]. The discrepancy might be caused by compensated effects induced by activation of p38 MAPK and JNK1/2 in ERK1/2 deletion mice treating with angiotensin II for 14 days. In our present study, treatment with Nox1/4 inhibitor GKT137831 or selective EGFR inhibitor AG1478 resulted in marked reduction in ERK1/2 activity in the left ventricle of SHRs. Hence, Nox1/4 inhibitor GKT137831 inhibits hypertensive cardiac hypertrophy via suppressing the ROS-EGFR pathway and subsequent ERK1/2 activation.

Cardiac inflammation is closely related to activation of Akt and ERK1/2. IL-6 increases Akt activity through formatting a heterohexameric complex consisting of two molecules each of IL-6, IL-6 receptor, and IL-6 receptor subunit *β* (gp130) [40]. Moreover, Akt activation promotes TNF-*α*-induced elevation in mRNA levels of IL-6, IL-1*α*, and IL-1*β* in cardiac fibroblasts and cardiac hypertrophy in primary cardiomyocytes [41, 42]. Our previous findings indicated that the Akt-mTOR signaling induces isoproterenol- and TNF-*α*-induced upregulation of expression of proinflammatory cytokines IL-1*β*, IL-6, and TNF-*α* in the hypertrophic myocardium through NF-*κ*B activation [14]. ERK1/2 activation mediates gp130-induced cardiac hypertrophy and PE-induced TNF-*α* production [43, 44].

One main feature of hypertrophic cardiomyocytes is predominance of the immediate-early genes and fetal gene (often referred to as hypertrophic genes, such as ANP and BNP) program again. Akt activates phosphorylation of GATA4 through glycogen synthase kinase-3*β* (GSK-3*β*), a major effector of Akt/PKB, and subsequent nuclear exit of GATA4, thereby inhibiting agonist-induced protein synthesis and expression of hypertrophic genes (ANP and BNP) in cardiomyocytes [45–47].

Knockdown of ERK2 decrease BNP expression in cardiomyocytes stimulated with phenylephrine through regulating BNP promoter activity [37]. Our present findings showed that treatment with Nox1/4 inhibitor GKT137831 or selective EGFR inhibitor AG1478 reduced activities of Akt and ERK1/2, as well as mRNA levels of ANP and BNP. Collectively, inhibiting activation of Akt and ERK1/2 mediates the beneficial role of Nox1/4 inhibitor GKT137831 in hypertensive cardiac hypertrophy via blocking predominance of hypertrophic genes.

## 5. Conclusions

In conclusion, this study reveals the protective action of Nox1/4 inhibitor GKT137831 against hypertensive cardiac hypertrophy in SHRs, and Nox-deprived ROS regulated EGFR activation through positive feedback in the hypertrophic myocardium, and inhibition of the ROS-EGFR pathway mediates the beneficial effect of GKT137831 on hypertensive cardiac hypertrophy by preventing cardiac inflammation and activation of Akt and ERK1/2. These findings will provide additional details for Nox1/4 inhibitor GKT137831 to prevent hypertensive cardiac hypertrophy.

## Figures and Tables

**Table 1:** Effect of Nox1/4 inhibitor GKT137831 on echocardiographic and hemodynamic parameters in spontaneously hypertensive rats.

	Control	SHR	SHR+GKT137831
LVAWd (mm)	1.62 ± 0.18	2.34 ± 0.27^#^	1.87 ± 0.13^∗^
LVAWs (mm)	2.33 ± 0.22	2.76 ± 0.19^#^	2.43 ± 0.18^∗^
LVPWd (mm)	1.56 ± 0.13	2.27 ± 0.225^#^	1.98 ± 0.18^∗^
LVPWs (mm)	2.43 ± 0.27	2.96 ± 0.23^#^	2.58 ± 0.14^∗^
LVIDd (mm)	6.67 ± 0.53	6.97 ± 0.57	6.72 ± 0.50
LVIDs (mm)	4.35 ± 0.46	4.57 ± 0.59	4.47 ± 0.47
Fractional shortening (%)	36.42 ± 4.15	36.09 ± 4.57	33.02 ± 2.75
Ejection fraction (%)	64.35 ± 5.86	67.32 ± 5.36	62.05 ± 9.46
AoSP (mmHg)	124 ± 8.0	186 ± 9.7^#^	176 ± 9.8
AoDP (mmHg)	83 ± 6.1	108 ± 9.6^#^	102 ± 6.5
Heart rate (beats/min)	350.8 ± 21.7	333.4 ± 22.0	335.8 ± 43.7
*dp*/*dt* max (mmHg/s)	4.87 ± 0.19	3.53 ± 0.15^#^	4.75 ± 0.10^∗^
*dp*/*dt* min (mmHg/s)	−4.71 ± 0.17	−3.39 ± 0.10^#^	−4.63 ± 0.18^∗^

SHR represents spontaneously hypertensive rats. LVAWd: left ventricular anterior wall thickness during diastole; LVAWs: left ventricular anterior wall thickness during systole; LVPWd: left ventricular posterior wall thickness during diastole; LVPWs: left ventricular posterior wall thickness during systole; LVIDd: left ventricular internal diameter during diastole; LVIDs: left ventricular internal diameter during systole; AoSP: aortic systolic pressure; AoDP: aortic diastolic pressure; *dp*/*dt* max: the maximal rate of left ventricular pressure increase; *dp*/*dt* min: the maximal rate of left ventricular pressure decrease. Data are expressed as mean ± standard deviation, *n* = 10. One-way ANOVA followed by post hoc test was carried out for the statistical analyses. ^#^*P* < 0.05 vs. the control group; ^∗^*P* < 0.05 vs. the SHR group.

**Table 2:** Effect of selective epidermal growth factor receptor (EGFR) inhibitor AG1478 on echocardiographic and hemodynamic parameters in spontaneously hypertensive rats.

	Control	SHR	SHR+AG1478
LVAWd (mm)	1.59 ± 0.14	2.30 ± 0.22^#^	1.83 ± 0.23^∗^
LVAWs (mm)	2.30 ± 0.23	2.75 ± 0.27^#^	2.37 ± 0.16^∗^
LVPWd (mm)	1.62 ± 0.15	2.33 ± 0.22^#^	1.93 ± 0.25^∗^
LVPWs (mm)	2.42 ± 0.23	2.99 ± 0.27^#^	2.65 ± 0.16^∗^
LVIDd (mm)	6.70 ± 0.58	6.93 ± 0.61	6.54 ± 0.79
LVIDs (mm)	4.31 ± 0.56	4.45 ± 0.57	4.33 ± 0.65
Fractional shortening (%)	36.22 ± 4.55	35.79 ± 4.03	34.68 ± 6.78
Ejection fraction (%)	64.53 ± 5.47	67.73 ± 5.79	62.75 ± 8.97
AoSP (mmHg)	122 ± 7.0	182 ± 8.7^#^	177 ± 8.9
AoDP (mmHg)	78 ± 6.4	105 ± 7.6^#^	103 ± 5.6
Heart rate (beats/min)	350.7 ± 21.6	334.2 ± 21.2	379.7 ± 30.7
*dp*/*dt* max (mmHg/s)	4.93 ± 0.18	3.50 ± 0.17^#^	4.52 ± 0.16^∗^
*dp*/*dt* min (mmHg/s)	−4.75 ± 0.13	−3.42 ± 0.17^#^	−4.38 ± 0.25^∗^

SHR represents spontaneously hypertensive rats. LVAWd: left ventricular anterior wall thickness during diastole; LVAWs: left ventricular anterior wall thickness during systole; LVPWd: left ventricular posterior wall thickness during diastole; LVPWs: left ventricular posterior wall thickness during systole; LVIDd: LV internal diameter during diastole; LVIDs: LV internal diameter during systole; AoSP: aortic systolic pressure; AoDP: aortic diastolic pressure; *dp*/*dt* max: the maximal rate of left ventricular pressure increase; *dp*/*dt* min: the maximal rate of left ventricular pressure decrease. Data are expressed as mean ± standard deviation, *n* = 10. The analytical tests were implemented using one-way ANOVA followed by the post hoc test. ^#^*P* < 0.05 vs. the control group; ^∗^*P* < 0.05 vs. the SHR group.

## Data Availability

The data used to support the findings of this study are available from the corresponding authors upon request.

## Supplementary Materials

Supplementary Materials Table S1: effect of Nox1/4 inhibitor GKT137831 on systolic blood pressure in spontaneously hypertensive rats. Table S2: effect of selective epidermal growth factor receptor (EGFR) inhibitor AG1478 on systolic blood pressure in spontaneously hypertensive rats.
