# Supplementary material for: Inhibition of the ROS-EGFR Pathway Mediates the Protective Action of Nox1/4 Inhibitor GKT137831 against Hypertensive Cardiac Hypertrophy via Suppressing Cardiac Inflammation and Activation of Akt and ERK1/2
Source: Mediators Inflamm. 2020 Aug 4;2020:1078365. doi: 10.1155/2020/1078365 (PMC7424508; doi:10.1155/2020/1078365)
Supplement: Supplementary Materials — Table S1: effect of Nox1/4 inhibitor GKT137831 on systolic blood pressure in spontaneously hypertensive rats. Table S2: effect of selective epidermal growth factor receptor (EGFR) inhibitor AG1478 on systolic blood pressure in spontaneously hypertensive rats. [file 1078365.f1.docx]

|  |
| --- |

Table S1 Effect of Nox1/4 inhibitor GKT137831 on systolic blood pressure (SBP) in spontaneously hypertensive rats.

| SBP | 0 week | 4^th^ week |
| --- | --- | --- |
| Control | 114±6.8 | 120±8.9 |
| SHR | 164±8.4^#^ | 182+10.2^#^ |
| SHR+GKT137831 | 162±6.0 | 174±8.8 |

SHR represents spontaneously hypertensive rat. Data are expressed as mean ± standard deviation, n = 10 rats per group. The statistical analyses were performed by two-way ANOVA followed by post hoc test. ^#^*P*<0.05 vs. Control.

Table S2 Effect of selective epidermal growth factor receptor (EGFR) inhibitor AG1478 on systolic blood pressure (SBP) in spontaneously hypertensive rats.

| SBP | 0 week | 4^th^ week |
| --- | --- | --- |
| Control | 115±7.9 | 122±9.0 |
| SHR | 160±6.5^#^ | 180+9.8^#^ |
| SHR+AG1478 | 162±7.4 | 172±7.8 |

SHR represents spontaneously hypertensive rat. Data are expressed as mean ± standard deviation, n = 10 rats per group. The two-way ANOVA followed by post hoc test was used for the statistical tests. ^#^*P*<0.05 vs. Control.
